# Independent evolution of intracellular transposons from exogenous IAP-related retroviruses in a broad range of mammalian species

**DOI:** 10.1186/1742-4690-8-S1-A226

**Published:** 2011-06-06

**Authors:** Gkikas Magiorkinis, Aris Katzourakis, Robert Gifford, Robert Belshaw

**Affiliations:** 1 Department of Zoology, University of Oxford, Oxford, England, UK; 2 Aaron Diamond AIDS Research Center, Rockefeller University, New York City, New York, USA

## 

Endogenous retroviruses (ERVs) are the descendants of occasional germ line invasions by exogenous retroviruses. Following these invasion events, some ERV lineages have proliferated by viral reinfection whilst others have become retrotransposon-like, losing the env gene which is required for infectivity of viral particles. The processes by which intracellular retrotransposons evolve from exogenous retroviruses remain poorly understood. The IAP (Intracisternal A-type retroviral Particles) family of ERVs provides an excellent model group in which to study this phenomenon. First identified in the Mouse genome, this well-defined and relatively recent group of ERVs have been studied extensively in vivo. We mined 36 mammalian genomes for all IAP-like ERVs, finding a total of 4913 loci within 17 genomes, mostly in several rodent species. Using these data, and taking in account the known phylogeny of the hosts, we analysed how the integrity of the env gene is related to the proliferation of IAP lineages within the host genomes. We found that loss of env is associated with the expansion of these lineages; for example in the three largest expansions accounting for more than 50% of the IAP-like loci, env has suffered extensive degradation. Our findings indicate that large copy number in IAP lineages is associated with them becoming retrotransposon-like, a process which could also apply to other, poorly studied, ERV groups. We are currently trying to identify other factors that influence this process and also to see how env loss affects other processes such as host switching.

